# Laser-Structured Si and PLGA Inhibit the Neuro2a Differentiation in Mono- and Co-Culture with Glia

**DOI:** 10.1007/s13770-022-00497-7

**Published:** 2022-12-20

**Authors:** Despoina Angelaki, Paraskevi Kavatzikidou, Costas Fotakis, Emmanuel Stratakis, Anthi Ranella

**Affiliations:** 1 Institute of Electronic Structure and Laser, Foundation for Research and Technology- Hellas (IESL- FORTH), 711 10 Heraklion, Greece; 2 Department of Physics, University of Crete, 710 03 Heraklion, Greece

**Keywords:** Neuronal differentiation, Glia-neurons co-culture, Nano/micro topography, Laser structuring, Neural tissue engineering

## Abstract

**Background:**

The first step towards a successful neural tissue engineering therapy is the development of an appropriate scaffold and the *in vitro* study of the cellular response onto it.

**Methods:**

Here, we fabricated nano- and micro- patterned Si surfaces via direct ultrafast laser irradiation, as well as their replicas in the biodegradable poly(lactide-co-glycolide), in order to use them as culture substrates for neuronal cells. The differentiation of neuro2a cells on the Si platforms and their replicas was studied both in a mono-culture and in a co-culture with glial cells (Schwann—SW10).

**Results:**

It was found that the substrate’s roughness inhibits the differentiation of the neuronal cells even in the presence of the differentiation medium, and the higher the roughness is, the more the differentiation gets limited.

**Conclusion:**

Our results highlight the importance of the substrate’s topography for the controlled growth and differentiation of the neuronal cells and their further study via protein screening methods could shed light on the factors that lead to limited differentiation; thus, contributing to the long standing request for culture substrates that induce cells to differentiate.

**Supplementary Information:**

The online version contains supplementary material available at 10.1007/s13770-022-00497-7.

## Introduction

Since decades, the golden standard to repair injuries of the peripheral nervous system (PNS) is the use of autologous nerve grafts (autografts) [1]. However, this approach involves several drawbacks, such as morbidity at the donor site, limited availability of grafts and more importantly it rarely leads to the full recovery of the patient [2]. As a response to these limitations, neural tissue engineering (NTE) has emerged as an alternative approach, aiming at repairing injured nerves with the use of cells. NTE also encompasses the development of appropriate scaffolds that have the ability to support the cell growth and eventually to be implanted at the site of the injury. The scaffolds used in NTE, and generally in tissue engineering, play a very important role, as they can affect vital cell functions, such as their attachment, proliferation, morphology and differentiation [3]. A large variety of materials and fabrication methods has been used for the development of scaffolds for neural tissue applications, including photolithography and soft lithography, laser structuring, electrospinning, 3D printing, etc. [4].

Surface structuring via ultrafast laser pulses has been used for the development of NTE scaffolds, as it is a simple and high fabrication rate technique that can produce a variety of scaffolds depending on the irradiation conditions and the material used [5, 6]. Previous research had shown that laser microstructured Si substrates can support the adhesion and growth of different types of cells, i.e. fibroblasts [7], Schwann cells [8, 9], PC12 cells [10]. At the same time, the substrate’s topography affects the morphology and the differentiation of the cells. Specifically, it was shown that high roughness Si micro-cones did not support PC12 cell differentiation [10]. It has also been found that nano-rippled Si patterns inhibit the adhesion of SW10 cells [9, 11]. Moreover, the Si patterns have been replicated on the biodegradable poly(lactide-co-glycolide) (PLGA) via soft-lithography, showing that the SW10 cells attach and grow on the replicas in the same way as on the Si substrates [12].

Nervous tissue consists of neurons and supporting cells called glia. Therefore, when studying the properties of the NTE scaffolds, it is important to perform cultures that comprise both neuronal and glial cells, as they mimic the actual structure of the nervous system. Several studies have examined stem cell based co-cultures [13], but there is a limited number of co-cultures with cell-lines reported. However, it is generally accepted that the use of cell-lines is simpler, faster and free of ethical issues, while they could replace the use of stem cells for the indispensable *in vitro* pre-clinical step towards the development of the clinical therapy [14].

The cell-lines-based co-cultures of the PNS involve Schwann cells and a neuronal cell-line and are mostly used as models to examine the myelination or the demyelination process. Schwann cells are one of the two types of glial cells found in the PNS and their role is to form myelin sheaths that insulate the nerve fibers. Takaku et al. [15] co-cultured adult rat Schwann cells IFRS1 and NSC-34 motor neuron-like cells as a study-model for the pathogenesis of motor neuron diseases, while Ishii et al. [16] co-cultured differentiated neurons derived from an adult rat neural stem cell line 1464R or motoneurons derived from a mouse embryonic stem cell line NCH4.3 with IFRS1 Schwann cells to form a myelinating system *in vitro*. Other works have studied the myelination process [17] and the toxic effect of amiodarone hydrochloride as a cause of demyelination in the PNS [18] in co-cultures of the neural crest-derived pheochromocytoma cell line (PC12) and the immortalized Schwann cell line IFRS1.

However, the studies described above are performed on flat substrates and they do not take into consideration the fact that the natural environment of the cells is the extracellular matrix (ECM), offering to the cells a variety of topographical cues [19, 20]. A study reporting a co-culture of PNS cell-lines on a non-flat topography was performed by Lizarraga‐Valderrama et al. [21] who used aligned polyhydroxyalkanoate blend microfibres to co-culture neuronal NG108‐15 and RN22 Schwann cells and found that the number of the neuronal cells increased when co-cultured with RN22 cells, while the fibres guided their aligned growth. Other studies of co-cultures of PNS cells on non-flat substrates have been performed with a neuronal cell-line and primary Schwann cells (SCs) that are isolated from animals. For example, Daud et al. [22] studied the co-culture of NG108‐15 neuronal cells, a hybrid cell line from mouse neuroblastoma- and rat glioma cells, with SCs on aligned electrospun polycaprolactone fibres and observed increased neurite length in the co-culture compared to the corresponding mono-culture. The same cells, SCs and NG108-15 neuronal cells, were also co-cultured in a spheroidal culture with subsequent sprouting assay in collagen gel and a significant increase of neurite length compared to the conventional 2D culture was observed [23].

Neuro2a (N2a) cells, a murine neuroblastoma cell-line, can be differentiated to neurons. N2a cells have been studied in co-cultures with 32D cells, a myeloblast-like *cell-*line, as a proof of concept of a patterned co-culture composed of adherent and nonadherent cells [24]. Also, they have been studied in a co-culture with bone marrow stem cells (BMSCs) on a 3-D cryogel matrix to develop an in vivo model for the repair of a nerve gap in the sciatic nerve of rats [25]. N2a co-culture with mesenchymal stem cells (MSCs) led to significant increase in the average neurite length, compared to control conditions [26]. Finally, the co-culture of N2a and SW10 cells was attempted on a combined nano- and micro- topography in Si and it was shown that when the N2a cells are co-cultured with glia, they change their adhesion behaviour and they follow the adhesion pattern of the glia, adhering solely on the areas where the SW10 cells grow [11].

Taking this outcome one step further, in this work we study the differentiation of the N2a cells in a mono-culture and in a co-culture with SW10 cells on the nano- and micro- structured Si substrates and on their PLGA replicas. The neuronal cells attachment, spreading and differentiation were assessed in correlation to the substrate’s topography, material and the presence of glia. The goal of this study is twofold. On the one hand, knowing that the micro-cones topography affects the differentiation of PC12 cells [10], we aim to clarify if this is an intrinsic property of this specific cell line, or if it is a general conclusion valid for other cell types as well. We also explore the effect of the nano-topography on the differentiation of neuronal cells. On the other hand, knowing that the adhesion behaviour of N2a cells changes in co-culture with glia [11], we aim to study the process of N2a differentiation in co-culture. The ultimate goal is the development of a spatially defined, functional neuronal network that could guide the treatment of the peripheral nerve injury and could be also used for the development of silicon-based neural implants and neurochips.

## Materials and methods

### Fabrication and characterization of micro- and nano- laser structured Si substrates

**Fig. 1:**
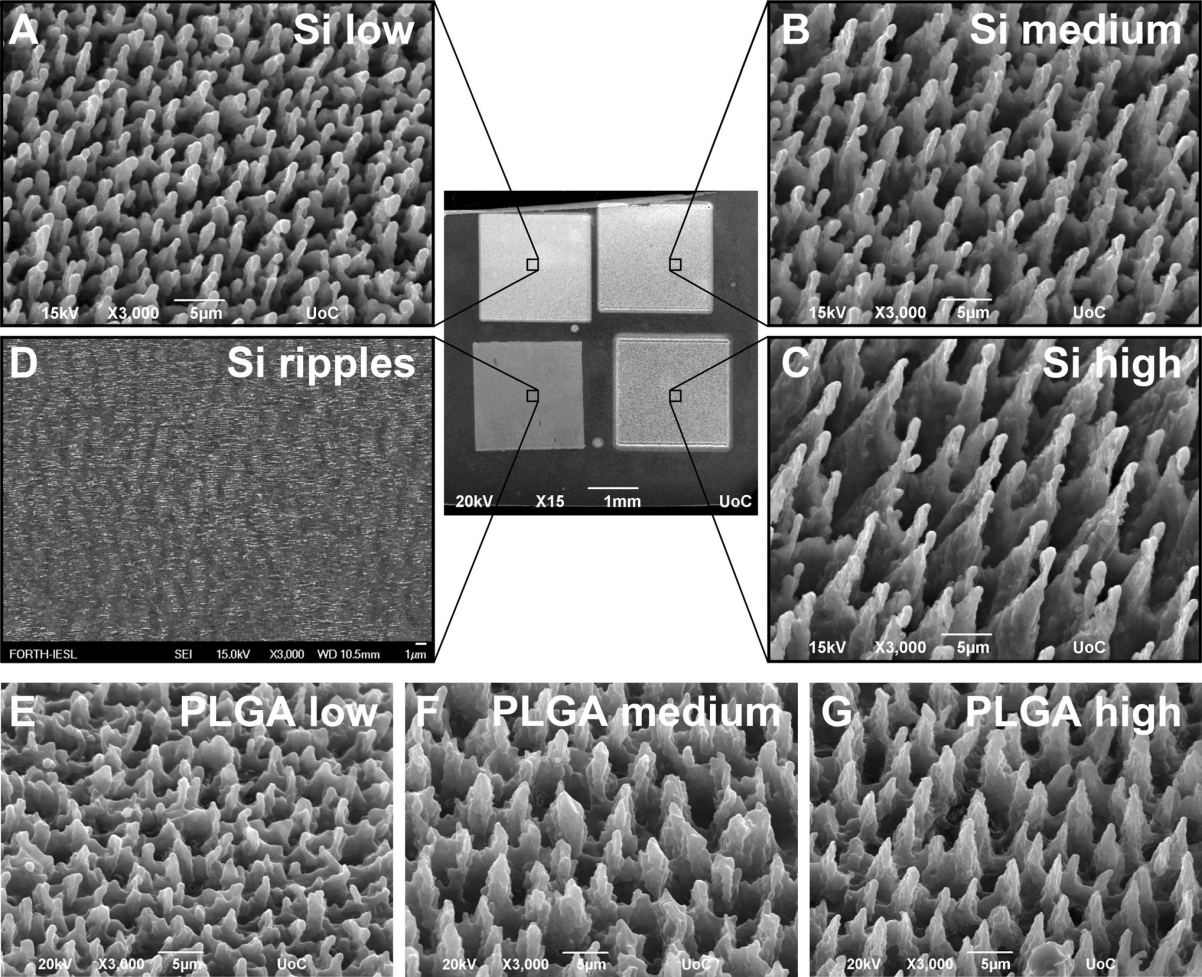
SEM images of the Si substrates with squares (size of 2000 × 2000  μm^2^ each) of **A** low, **B** medium and **C** high roughness micro-cones and **D** nano-ripples and of **E** low, **F** medium and **G** high roughness PLGA replicas

The fabrication of the micro- and nano- structured Si substrates was performed via an ultra-short pulsed laser of 1026 nm wavelength, delivering 170 fs pulses at a repetition rate of 1 kHz. A single crystal Si wafer was mounted on a high-precision X–Y translation stage, perpendicular to the laser beam. In aqueous environment and at low laser fluence (0.20 J/cm^2^), arrays of periodic ripples—of 146 ± 37 nm periodicity—were fabricated [9]. In reactive gas atmosphere (sulfur hexafluoride—SF_6_, pressure of 650 mbar) and with higher laser fluence, arrays of micro-cones (spikes) were structured on the Si surface. Squares of spikes were formed in three different laser fluences (0.42–0.72 J/cm^2^), each containing micro-cones of different size and density. Eventually, the Si substrates included four laser-patterned squares of different roughness (nano-ripples, low, medium and high roughness micro-cones) and of 2000 × 2000 μm size, as shown in Fig. 1.

The morphological characteristics of the structured Si substrates were assessed via scanning electron microscopy (SEM), as described in previous works [9, 10]. SEM observation was performed on a JEOL JSM-6390LV SEM (JEOL Ltd., Tokyo, Japan) with an acceleration voltage of 15 kV. For the image analysis, Fiji ImageJ [27], an image processing software, was used in order to determine the geometrical characteristics of the micro-cones and the nano-ripples.

### Si substrate treatment

Following the irradiation, the patterned Si substrates were immersed in hydrofluoric acid for ~ 1 h, rinsed in nanopure water and dried with nitrogen flow. The substrates were then sterilized in an autoclave and no additional coating was used for the *in vitro* cultures.

### Fabrication of polymeric replicas

The replicas of the laser-patterned micro-structures were produced via a two steps replication process. Initially, the negative replicas of the three different roughness (low, medium and high) of micro-cones were fabricated in elastomeric PDMS (SYLGARD 182, Dow Corning, Midland, MI, USA). Liquid PDMS pre-polymer consisting of a “base” and “curing agent”, typically mixed in a 10:1 w:w ratio, was poured onto each substrate [28]. In order to remove possibly residual air bubbles, the PDMS-coated Si substrates were placed in a vacuum oven. After heating at 80 °C for 2 h, the negative molds of the original micro-patterns were peeled off the Si substrates. Using the PDMS negative molds, replicas of the initial morphology were produced in PLGA. Specifically, PLGA (lactide:glycolide 65:35, MW 40–75 k) polymeric solution of 1:10 (Code No: P2066, Sigma-Aldrich, St. Louis, MO, USA) in dichloromethane (DCM) was prepared. The PLGA solution was magnetically stirred for 2 h in a chemical hood at room temperature and then poured onto each PDMS negative mold. After having let the solvent of the PLGA-coated PDMS mold to evaporate for 24–48 h in-18 °C (refrigerator), the molds were cooled in 4 °C for 2 h. Finally, the PLGA replicas were carefully peeled from the PDMS negative mold with a pair of tweezers [12].

### Cell cultures

The neuro2a cell-line was used to study the effect of the micro- and nano- topography of laser-patterned Si substrates and their polymeric replicas on the differentiation of the neuronal-like cells in mono-culture and in co-culture with glial cells (SW10). Schwann cells are the principal glia of the PNS, while the N2a cells were originally derived from a spontaneous tumor in an albino mouse neuroblastoma and can be differentiated into cells with many properties of neurons.

The cells were cultured in Dulbecco's modified eagle's medium (DMEM; Gibco, Carlsbad, CA, USA) supplemented with 10% fetal bovine serum (FBS; Bioseral, CBINSIGHT, New York, NY, USA), and 1% antibiotic (Pen-Strep) solution (GIBCO, Invitrogen, Carlsbad, CA, USA) and incubated at 37 °C in a 5% CO_2_ atmosphere.

All cultures were repeated at least three times and each time flat Si substrates, as well as the conventional tissue culture plastic (TCP) disks were used as the control substrates.

### N2a differentiation

For the N2a cells differentiation experiments, 6·10^4^ cells/ml were seeded on the silicon scaffolds, PLGA replicas and tissue-culture plastic disks and cultured for 1 day with DMEM 10% FBS and for 3 days with DMEM without FBS and with 10 μM retinoic acid (RA; Sigma-Aldrich), until fixation for further analysis. As an alternative differentiation method, FBS deprivation with 300 μM cyclic adenosine monophosphate (cAMP; Sigma-Aldrich) for 3 days, after culture for 1 day with DMEM 10% FBS, was used. TCP disks were also seeded for 4 days with DMEM 10% FBS and a change of medium was performed after the first day of culture.

The optimal concentrations of the differentiation media were determined after preliminary experiments, where RA concentrations between 0,1 μΜ and 20 μΜ and cAMP concentrations between 10 and 1 mM were tested.

### SW10 with differentiation media

In order to study the effect of the differentiation media on the SW10 cell cultures, the same culture conditions as in the case of the N2a differentiation experiments were utilized. 6·10^4^ cells/ml were seeded on the silicon scaffolds and cultured for 1 day with DMEM 10% FBS and for 3 days with DMEM without FBS and with 10 μM retinoic acid. Moreover, the effect of cAMP on SW10 cells was studied and therefore cultures with FBS deprivation and 100, 300, 500 and 1000 μM of cAMP for 3 days, after 1 day with DMEM 10% FBS were performed.

### SW10 & N2a co-culture with cAMP

In order to study the differentiation of the N2a cells in a co-culture with SW10 cells, 4,5·10^4^ cells/ml of each kind were simultaneously seeded on the Si substrates, PLGA replicas and tissue culture plastic disks. The cells were cultured for 1 day with DMEM 10% FBS and for 3 days with DMEM without FBS and with 300 μM cAMP until fixation for further analysis.

### SEM analysis of cultured cells' morphology

The adhesion and the differentiation of the cultured cells were assessed via SEM imaging and subsequent analysis. Before the SEM examination, the Si samples were subjected to a critical point drying (CPD) process, a widely used fixation method for the dehydration of biological specimens. More specifically, the Si samples were washed with 0.1 M sodium cacodylate buffer (SCB) and then incubated in the same solution for 10 min. After repeating this step twice, the Si samples were fixed with 2% glutaraldehyde, 2% formaldehyde in 0.1 M SCB fixative buffer for 30 min at 4 °C. All samples were then washed twice (for 10 min each time) with 0.1 M SCB at 4 °C and dehydrated by immersion in serially graded ethanol solutions (30–100%) and incubated finally for 10 min in dry 100% ethanol. Lastly, the Si samples were dehydrated in a Baltec CPD 030 critical point dryer and sputter-coated with a ~ 14 nm gold layer in a Baltec SCD 050 sputter coater.

For the PLGA replicas, CPD cannot be used since it deforms the polymer, so, after an optimization process, a hexamethyldisilizane (HDMS) protocol was established. After the dehydration steps with ethanol (EtOH), EtOH:HDMS solutions (50:50) were used for specific time points for all the replicas, and then the same procedure was repeated with HDMS solutions. Finally, the replicas were left to dry at room temperature overnight.

### Immunocytochemistry

A series of specimens were immunostained in order to study the cell viability, adhesion and differentiation. The culture medium was removed, the samples were washed with PBS and then the cell cultures were fixed with 4% paraformaldehyde for 15 min. Afterwards, they were permeabilized with immersion in PBS—Triton 0.1% for 5 min and blocked in PBS—BSA 2% (blocking solution) for 30 min.

For the staining of actin fibers in SW10 cells, the cells were incubated with Alexa Fluor® 568 Phalloidin (Invitrogen, Thermo Fisher Scientific, Waltham, MA, USA) (1:500 in PBS–BSA 1%) for 1 h. After wash with PBS, nuclei were stained with DAPI and the substrates were mounted on coverslips.

In the co-culture system, the Schwann cell-specific protein S100 was labeled with rabbit anti-S100b (1:100 in blocking buffer; Abcam, RabMab ab52642) primary antibody overnight at 4 °C. After 2–3 washes with PBS, specimens were incubated for 2 h at room temperature with Alexa Fluor® 546 goat anti-rabbit IgG (Invitrogen, Thermo Fisher Scientific) secondary antibody, diluted 1:500 in PBS–BSA 1%.

To identify differentiated N2a cells, the neuron-specific class III β-tubulin marker was detected using the MAB163 monoclonal antibody. Thus the cells were incubated with the MAB1637 monoclonal antibody (1:500 in PBS–BSA 2%; Millipore, MA, USA) and subsequent labelling with goat–anti-mouse FITC conjugate secondary antibody (1:200 PBS–BSA 2%; Biotium, Fremont, CA, USA).

The cells’ cytoskeletons on the TCP control disks for the experiments on PLGA were stained additionally in grey with phalloidin 680 (1:1000).

After wash with PBS, nuclei were stained with DAPI and the substrates were mounted on coverslips.

#### Image processing and statistical analysis

A Laser Scanning Spectral Confocal Microscope (Leica TCS SP8, Wetzlar, Germany) was used for the observation of the immunostained substrates. The image processing software Fiji ImageJ [27] was used to perform the quantitative analysis of the SEM and fluorescence microscopy images.

The number of cells was determined by counting cell nuclei stained with DAPI. The number of nuclei was assessed with the Fiji ImageJ "Cell Counter" plugin. The number of differentiated cells was determined by visual examination of the image field. Cells with at least one neurite of the same length or longer than the cell body diameter were considered as differentiated. The differentiation ratio was defined as the number of differentiated cells divided by the total number of cells in a specific area, expressed as a rate per 100. The mean length of the longest neurite was calculated by measuring the distance between the edge of the cell soma to the neurite tip for the longest neurite of each differentiated N2a cell in the studied area (using the Line Selection Tool and the Measure function to record the line length).

The results represent the means of at least three different experiments.

For further statistical analysis, the data were subjected to one way ANOVA followed by Tukey test for multiple comparisons between pairs of means.

## Results

### Fabrication and characterization of micro- and nano- structured Si substrates and their PLGA replicas

The Si substrates were fabricated via direct ultra-fast laser structuring. The morphology of the resulting structures, their size and density, depends on the laser fluence and the irradiation environment (i.e. liquid, vacuum or reactive gas) [7, 9, 10]. More specifically, irradiation in aqueous environment at low laser fluence (~ 0.20 J/cm^2^) gives rise to nano-ripples. By increasing the laser fluence, the irradiation leads to the development of micro-cones and when the texturing takes place in a reactive gas atmosphere e.g. sulfur hexafluoride SF_6_, the resulting micro-cones are better-shaped and of higher aspect ratio compared to structuring in vacuum. Three types of micro-cones (low, medium and high roughness) with different geometrical characteristics were developed. A SEM image of the laser-structured Si substrates used in the present research is shown in Fig. 1, accompanied by high-magnification views of the nano-ripples and the micro-cones of the three different roughness values.

The geometrical characteristics of the Si structures have been extensively studied in earlier works [9, 10]. In brief, the spikes are more arbitrary at low laser fluence, as their cross-section is arbitrary-shaped and they display a broad range of orientation angles (the angle between the major axis of the elliptic cross-section of the spike with respect to the vertical y-axis of the SEM image plane). When the fluence increases, the spikes are more ordered, as their cross-section becomes almost elliptic and they are mostly aligned in parallel. Moreover, the spikes become larger (higher and with bigger cross-sections), resulting in a longer interspike distance and a lower density (number of spikes per cm^2^) (Table 1) [8, 10]. All four different laser-patterned surfaces (i.e. nano-ripples, low, medium and high roughness spikes) become hydrophilic after the autoclave sterilization [11], therefore we can safely postulate that the underlying topography is the only parameter that changes during the experimental studies performed.

**Table 1:** Geometrical characteristics of the Si micro-cones

Si spikes roughness	Density (10^6^ spikes/cm^2^)	Height (μm)	Major axis (μm)	Minor axis (μm)	Inter-spike distance (μm)
Low	13.15 ± 3.96	3.59 ± 0.35	2.82 ± 0.28	1.51 ± 0.15	3.17 ± 0.71
Medium	6.06 ± 0.96	6.94 ± 0.58	4.03 ± 0.39	2.11 ± 0.21	4.68 ± 0.94
High	3.45 ± 0.32	13.06 ± 1.32	6.30 ± 0.68	3.68 ± 0.56	6.67 ± 1.27

The replicas of the laser-patterned micro-spikes were produced via soft-lithography with the use of a negative PDMS mold. Their geometrical characteristics have been previously determined [12]. In short, as expected, they follow the same size trend as the Si spikes, i.e. their height increases with the laser fluence, while their density is the lowest in the high-roughness structures. The direction of the replicated spikes lies at the area of zero degrees for the medium- and high-roughness PLGA micro-cones, while the low roughness substrate showed a lower directionality at the area close to 52 degrees. Moreover, increasing the roughness of the PLGA replicas surface decreased the hydrophilicity. SEM images of the replicated in PLGA spikes are shown in Fig. 1.

### Differentiation studies

Previous studies have shown that the N2a cells adhere both on the nano-ripples and on all three micro-conical Si substrates of different roughness. On the contrary, SW10 cells do not adhere on the nano-ripples, but they adhere on the spikes of different roughness. However, when N2a are co-cultured with SW10 cells, they change their adhesion behaviour and they also adhere exclusively on the spikes, following the adhesion pattern of the glial cells [11]. In another study it was shown that SW10 cells attach strongly and proliferate well on the PLGA spikes’ replicas, while they also align along the direction of the spikes [12].

In the present work, a differentiation study was performed to all four different laser-patterned surfaces (i.e. nano-ripples, low, medium and high roughness spikes), as well as the spikes’ replicas, both for the mono-cultures and the N2a and SW10 co-culture. For the mono-cultures in Si, two alternative differentiation methods were used; FBS deprivation with RA and FBS deprivation with cAMP. In both cases the cells were cultured for one day with 10% FBS medium and then the medium was changed to growth medium without FBS.

### Differentiation study of N2a cells

**Fig. 2:**
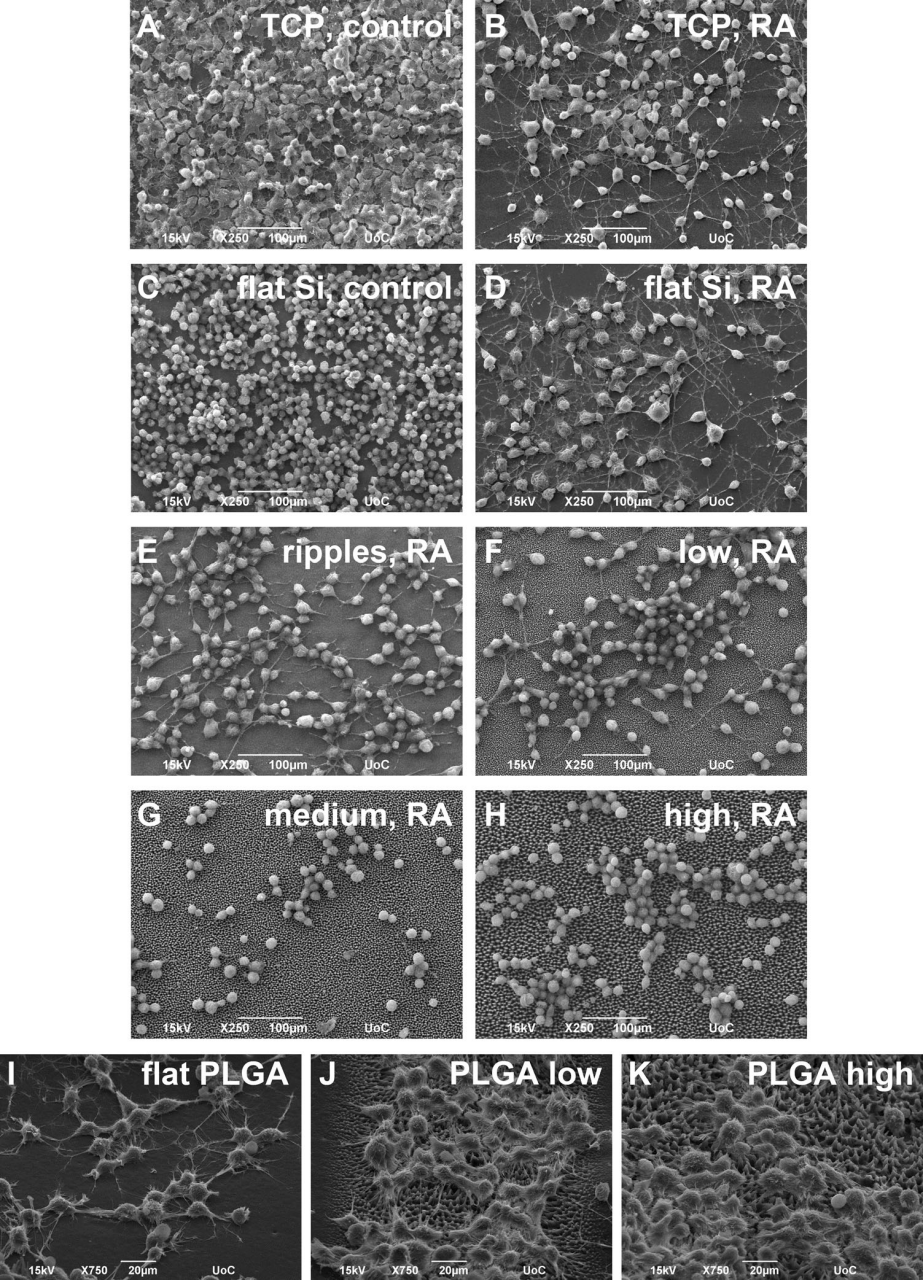
SEM images of the 1 + 3 days N2a culture (6·10^4^ cells/ml). On standard TCP disks **A** without RA (control substrate) and **B** with 10 μΜ RA. Without RA on the flat Si substrate (control substrate) **C**, with 10 μΜ RA on the: **D** flat Si substrate, **E** nano-ripples, **F** low roughness micro-cones, **G** medium roughness micro-cones, **H** high roughness micro-cones, **I** flat PLGA, **J** low roughness and **K** high roughness PLGA replicas

**Fig. 3:**
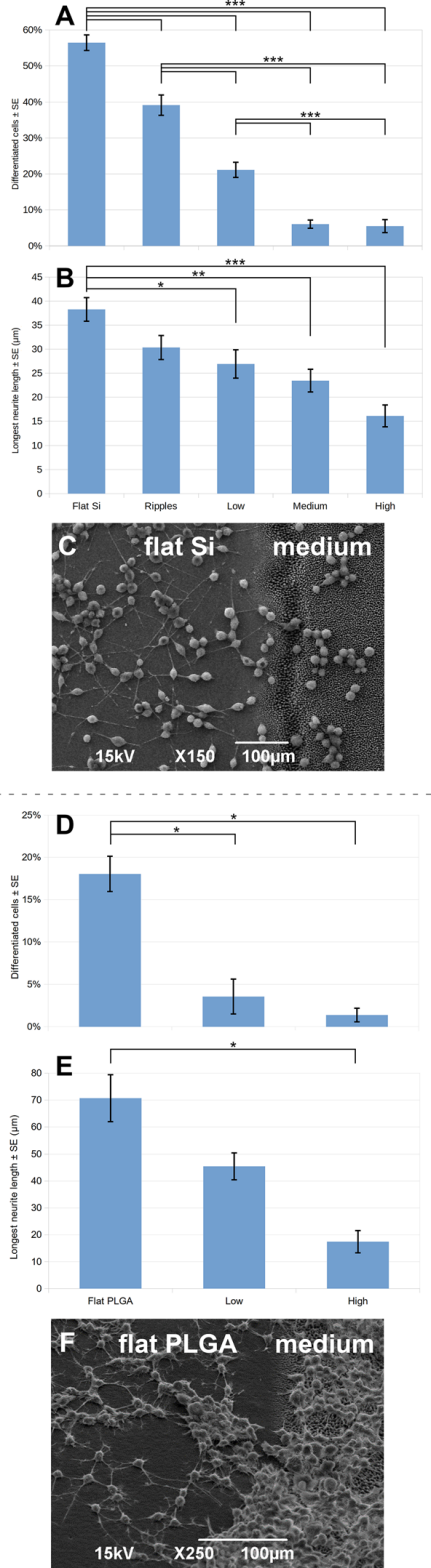
**A** and **D** Percentage and **B** and **E** longest neurite length of differentiated N2a cells cultured for 1 + 3 days on **A** and **B** laser patterned Si substrates and **D** and **E** their PLGA replicas. The results are expressed as **A** and **D** percentage ± standard error of the mean (SE) and **B** and **E** μm ± SE. The data were subjected to ANOVA followed by Tukey test for multiple comparisons between pairs of means. The difference between **A** the percentage of differentiated N2a cells growing on any kind of Si surface is statistically highly significant (***p < 0.001), except the difference between the medium and high roughness substrates, that is non-significant and **B** the length of the longest neurite of differentiated N2a cells growing on flat Si surface and low roughness spikes is statistically significant (*p < 0.05), statistically very significant (**p < 0.01) for medium roughness spikes and statistically highly significant (***p < 0.001) for the high roughness spikes. The difference between **D** the percentage of differentiated N2a cells growing on flat PLGA, low- and high- roughness replicas and **E** the length of the longest neurite of differentiated N2a cells growing on flat PLGA and high roughness replicas is statistically significant (*p < 0.05). **C** and **F** SEM images of the 1 + 3 days N2a culture (6·10^4^ cells/ml) with 10 μΜ RA on the interface between the **C** flat Si and the medium roughness spikes and **F** the flat PLGA and the medium roughness replicas

When the culture medium was changed after the first day *in vitro* and was replaced with medium without FBS and with 10 μM of RA, the N2a cells started differentiating and they grew long neurites, forming a network on the flat Si and the flat PLGA. A similar situation was observed for the N2a culture on the Si nano-ripples; the cells started the differentiation process and long axons were developed. As far as it concerns the Si micro-cones and their replicas, the results obtained for the N2a cell-culture were strikingly different from the previous ones and an interesting phenomenon took place. On the low roughness spikes, a limited number of axons was developed, while on the medium and high roughness spikes, the N2a cells remained in the undifferentiated state. Remarkably, the roughness of the substrate inhibits the differentiation, even in the presence of the differentiation medium, the retinoic acid. Figure 2 shows SEM images of the N2a cells differentiation on flat and laser-patterned Si, as well as on flat PLGA and PLGA replicas. An image of N2a culture without the use of any differentiation medium, as well as images of the culture on the standard tissue culture plastic disks, are also shown for comparison. The quantification of this interesting finding is shown in Fig. 3, where the percentage of the differentiated N2a cells on each laser patterned Si substrate is shown, as well as their longest neurite length. The same results are shown for the flat PLGA and the low and high roughness PLGA spikes’ replicas. The quantification for the TCP disks is not shown, as it is similar to the flat substrate for each material with a non-statistically significant difference. The percentage of differentiated N2a cells is defined as the number of cells with at least one neurite with a length equal or longer to the cell body diameter compared to the total number of cells that adhered on the surface, while the longest neurite length is defined as the mean length of the longest neurite per cell for cells with at least one identified neurite. In addition, SEM images of the N2a culture with 10 μΜ RA on the interface between the flat Si and the medium roughness spikes, as well as the flat PLGA and the medium roughness replicas are shown for conspicuousness reasons.

The results show that the percentage of differentiated cells decreased drastically on the Si laser-patterned surfaces. The 56.45 ± 2.15% of differentiated N2a cells on the flat Si fell to 39.18 ± 2.82% on the nano-ripples and followed further reduction to 21.14 ± 2.12% on the low-roughness spikes, reaching 6.05 ± 1.14% and 5.49 ± 1.83% on the medium and high-roughness spikes respectively (Fig. 3A). The neurite length of the differentiated cells followed a similar trend, with their length diminishing dramatically as the roughness of the surface was increasing; from 38.27 ± 2.45 μm on the flat Si to 16.09 ± 2.27 μm on high roughness spikes (Fig. 3B). Regarding the PLGA, the percentage of differentiated N2a cells on the flat was 18.04 ± 2.09%, while it fell to less than 5% for the different roughness of replicas (Fig. 3D). The neurite length on the flat PLGA was 70.69 ± 8.72 μm, showing an important difference from the low-roughness replicas (45.48 ± 4.98 μm), and it diminished further on the high-roughness replicas, reaching 17.45 ± 4.11 μm (Fig. 3E).

By comparing the percentage of differentiated cells on the two materials, we deduce that Si is more favorable for the differentiation of N2a than PLGA. However, on the PLGA replicas the N2a cells form longer neurites than on Si. It should be highlighted though that the neurites’ length eventually decreases almost to the same length on both the high-roughness Si and PLGA (16.09 ± 2.27 μm and 17.45 ± 4.11 μm respectively). In conclusion, on both materials the topographical cues negate the effect of the differentiation medium.

The same experiments were repeated with the use of an alternative differentiation medium, the cAMP. For the Si substrates, as well as for their PLGA replicas, the results obtained were similar to the ones with the RA; the N2a cells differentiated and created long neurites on the flat Si and the flat PLGA, but even the slightest roughness of the substrate inhibited their differentiation (Supplementary Fig. S1). Hence, we conclude that the substrate’s roughness runs contrary to the differentiation medium and manages to eliminate its effect on the cells; i.e. when the N2a cells are seeded on a rough substrate, they do not differentiate even in the presence of differentiation media (RA or cAMP).

### Differentiation study of SW10 and N2a cells in co-culture

Before attempting the differentiation of N2a cells in a co-culture with SW10, we conducted an *in vitro* culture of SW10 cells with the differentiation media. When the culture medium was changed after the first day and it was replaced with medium without FBS and with 10 μM of RA, the SW10 cells did not survive. Therefore, an alternative differentiation method was attempted and instead of RA, 300 μM of cAMP were used. Supplementary Fig. S2 shows the SW10 cells growing on flat Si in growth medium with FBS and in FBS deprived medium with RA or with cAMP. In the presence of cAMP, the SW10 cells grew normally on the flat Si, up to the concentration of 500 μM. However, as the roughness of the substrate increased, their attachment and growth was reduced and the cells remained round. Supplementary Fig. S3 shows the number of SW10 cells growing on flat, rippled, low, medium and high roughness Si substrates in the presence of cAMP. Consequently, for the co-culture of SW10 and N2a cells the differentiation method with 300 μM cAMP was used both for the cultures on Si substrates and on their PLGA replicas.

**Fig. 4:**
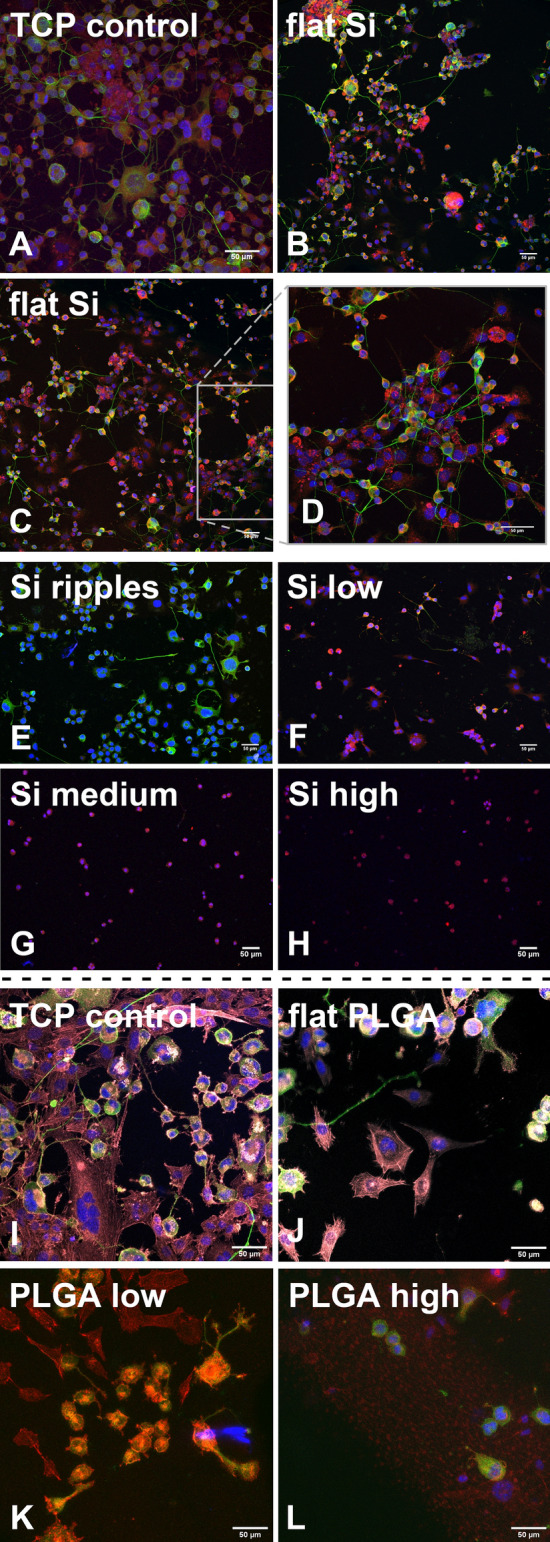
Confocal images of the 1 + 3 days N2a and SW10 co-culture with 300μΜ cAMP on the: **A** TCP disk (control), **B**–**D** flat Si, **E** rippled, **F** low, **G** medium and **H** high roughness Si substrates and **I** TCP disk (control), **J** flat PLGA, **K** low and **L** high roughness PLGA replicas. In red the S100 positive SW10 cells, in green the N2a neurites showing neuron-specific class III β-tubulin (stained with Tuj-1) and in blue the cells nuclei stained with DAPI. The cells’ cytoskeletons on the TCP control disks for the PLGA experiments (I & J) were stained additionally in grey with phalloidin 680

**Fig. 5:**
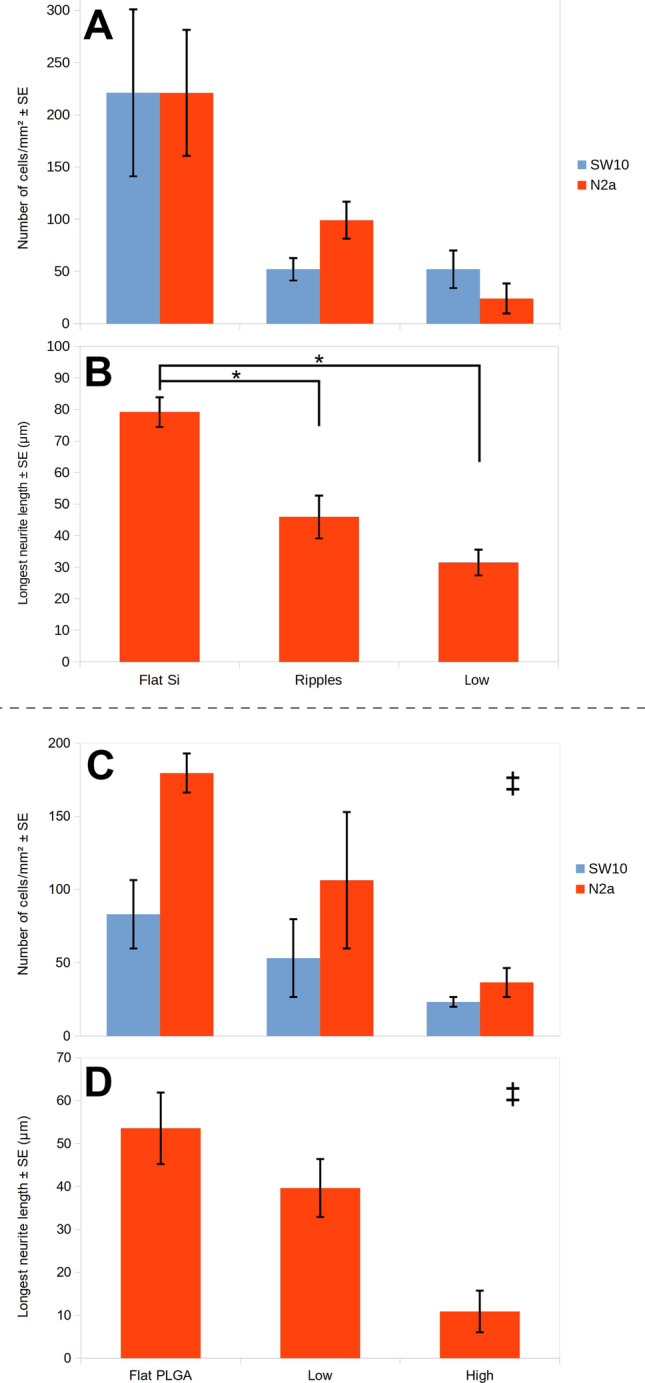
**A** and **C** Numbers of N2a and SW10 cells and **B** and **D** longest neurite length of the differentiated N2a cells co-cultured with SW10 for 1 + 3 days days on **A** and **B** flat Si, nano-ripples and low roughness micro-cones and on **C** and **D** flat PLGA, low and high roughness PLGA replicas with 300 μM cAMP. The results are expressed as **A** and **C** cells/mm^2^ ± standard error of the mean (SE) and **B** and **D** μm ± SE. The data were subjected to ANOVA followed by Tukey test for multiple comparisons between pairs of means. For **A** the results were not statistically significant (*p* > 0.05). For **B** the difference between the length of the longest neurite of differentiated N2a cells growing on flat Si surface and ripples or low roughness spikes is statistically significant (*p < 0.05). **‡** For **C** and **D** the difference between all groups is statistically significant (*p < 0.05)

Figure 4 shows the SW10 and N2a cells co-culture on TCP disks and on flat and patterned Si and PLGA in the presence of cAMP. On the flat Si, the Schwann cells exhibited good attachment and outgrowth, while the N2a cells developed long neurites (79.21 ± 4.69 μm, Fig. 4B–D). The quantitative analysis showed that a similar number of the two kinds of cells attached to the flat Si in the presence of cAMP (Fig. 5A). On the nano-ripples, the Schwann cells showed very limited attachment, while the N2a cells differentiated, but they developed shorter neurites than on the flat Si (45.96 ± 6.81 μm, Fig. 4E). On the micro-cones areas of the three different roughness, both the SW10 and the N2a cells followed the same adhesion and differentiation pattern as in the mono-culture with cAMP, i.e. we observe low number of differentiated N2a cells on the low roughness substrate (Fig. 4F), while there are no differentiated cells on the medium and high roughness substrates (Fig. 4G, H). The SW10 cells remained round on the medium and high roughness substrates and did not exhibit their typical bipolar or flattened morphology. Moreover, as the cells remained round, we can not make the distinction between the two kinds of cells and only the total number of cells attaching on a specific area for the medium and high roughness substrates can be provided. Therefore, in Fig. 5A the average number of Schwann and N2a cells adhering only on the flat, rippled and low roughness Si substrates in the presence of cAMP is shown. Accordingly, the longest neurite length (the mean length of the longest neurite per cell for cells with at least one identified neurite) of the N2a cells co-cultured with SW10 in the presence of cAMP for the flat Si, ripples and low roughness micro-cones is shown in Fig. 5B.

On the flat PLGA, the SW10 cells attached and grew well. Moreover, the N2a cells developed long neurites (53.58 ± 8.34 μm, Fig. 4J). On the low roughness replicas, the Schwann cells attached, while the N2a cells differentiated, but they developed shorter neurites than on the flat PLGA (39.63 ± 6.74 μm, Fig. 4K). On the high roughness replicas, there is very limited differentiation and the neurites length diminished further, reaching 10.89 ± 4.86 μm (Fig. 4L). In Fig. 5C the average number of Schwann and N2a cells adhering on the flat, low and high roughness PLGA replicas in the presence of cAMP is shown. In all three different PLGA substrates, the number of N2a cells adhering in the presence of cAMP is higher than the number of SW10 cells adhering. This is probably due to the fact of the cessation of proliferation that occurs before the formation of the first myelin wrap in the presence of cAMP [29]. The longest neurite length of the N2a cells co-cultured with SW10 in the presence of cAMP for the flat PLGA, low and high roughness replicas is shown in Fig. 5D.

By comparing the number of cells adhering on the two materials in co-culture in the presence of cAMP, we observe that it is similar for both the Si and the PLGA and that for both cases the number of cells decreases as the roughness increases. The N2a neurites length also diminishes with the increasing roughness, drawing the conclusion that the roughness of the substrate limits the effect of the differentiation medium.

## Discussion

Numerous studies have reported that nerve cells respond to underlying topography and consequently their attachment, proliferation, morphology and differentiation are drastically affected [3]. Neuronal differentiation has been studied in correlation with the topography of the culture substrate mostly on neural stem cells (NSC) [30] and PC12 cells [10, 31–34]. However, only few studies have explored the response of N2a cells on non-flat topographies and moreover their co-culture with glial cells has been hardly attempted before. Such a co-culture can be of great interest, as it encompasses both the glial and the neuronal-like cells of the PNS. In the present work, nano- and micro- patterned Si scaffolds were fabricated via ultra-fast laser irradiation and they were then replicated on PLGA via soft lithography. They were subsequently investigated as platforms for the differentiation of N2a cells in mono- and in co- culture with glia.

It was shown that the substrate’s roughness inhibits the differentiation of the neuronal cells even in the presence of the differentiation medium, for both the semiconductor and the polymeric material. In particular, the higher the roughness is, the more the differentiation gets limited, i.e. fewer differentiated cells and shorter neurites are observed. This outcome is in good agreement with the studies by Simitzi et al. [10] who have reported that high roughness Si micro-cones did not support PC12 cell differentiation. Therefore, based on our results, we could state that the inhibition of the neuronal-like cells differentiation is not an intrinsic property of a specific type of cells, as two different cell types (PC12 and N2a) demonstrated the same response, i.e. their differentiation was inhibited due to the topography of the culture substrate. This lets us presume that the topography of the substrate plays a more important role for the differentiation of the neuronal-like cells, than the presence of the differentiation medium. Since the same results were obtained not only for two different substrate’s materials (Si and PLGA), but also for two different differentiation media (RA and cAMP), this assumption can be further supported.

Here, we also examined the differentiation of N2a cells on rippled Si and we found that even this nano-topography (with 146 ± 37 nm periodicity) has the ability to limit the cells differentiation, i.e. from almost 57% of differentiated N2a cells on the flat Si to 39% on the nano-ripples (Fig. 3A). Thus reinforcing further the conclusion that the topographic cues influence the neuronal-like cells differentiation. The same effect has previously been observed on patterns of nano -pillars and -pores, having dimensions comparable with but slightly larger than that of filopodia (~ 100–150 nm), which limited the PC12 neurite outgrowth both in number and length [34].

Furthermore, it is interesting to compare our results with a relevant study with N2a cells. Specifically, Beduer et al. have reported a N2a differentiation rate close to 100% on PDMS micrometric grooves comprising lines and spaces with line width of 20 μm and microgrooves of 25 μm depth [35]. Given that the size of N2a cells is 14–17 μm in height and 15–25 μm in lateral dimensions [36], we could deduce that a pattern that is larger or comparable to the cell dimensions (i.e. the 20 μm wide and 25 μm deep PDMS grooves) seems to favor the N2a differentiation, while a pattern that is smaller than the cell dimensions (i.e. the Si micro-cones, whose inter-cone distance ranges from 3.17 ± 0.71 μm for the low roughness micro-cones to 6.67 ± 1.27 μm for the high roughness and their height is 3.59 ± 0.35 μm for the low roughness, reaching 13.06 ± 1.32 μm for the high roughness) inhibits the differentiation. This conclusion is also valid for the PLGA micro-cones replicas, as their height varies from 3.06 ± 0.40 μm for the low roughness structures to 10.55 ± 1.10 μm for the high roughness [12]. It is worth stating here that there is a slight difference between the fabricated topography (Si substrates) and the replicated topography (PLGA replicas) in terms of the spikes’ height due to the different stages of the soft lithography process. In conclusion, we could hypothesize that on the Si micro-cones and their PLGA replicas the cells did not have the necessary space in order to elongate, develop extensions and finally differentiate. This reinforces the assumption that in order to develop mature focal adhesion points the N2a cells require topographical cues in the same order of magnitude with their own size.

When the Schwann cells were cultured in the presence of retinoic acid, they did not survive and an alternative differentiation method with cAMP was used for the co-culture experiments. However, it is generally recognized that RA plays an important role during the development of the nervous system as a potent regulator of morphogenesis, cell growth and differentiation, while it has been shown that RA is also a potent inhibitor of peripheral nervous system myelination [37].

In the presence of cAMP, the SW10 cells grew normally on the flat Si, but, as the roughness of the substrate increased, their attachment and growth was reduced and the cells remained round. Schwann cells (SCs) are very responsive to cAMP during their lifetime, as their survival, lineage specification, proliferation and differentiation into myelin-forming cells require cAMP signaling. SCs respond to cAMP by transiting from an immature (proliferative) to a differentiated post-mitotic (growth arrested) state and the activation of cAMP signal transduction in SCs accelerates and greatly enhances myelin formation *in vitro* [38]. On that account, the presence of cAMP explains the reduced number of SW10 cells attached on the Si substrates, but their response to the micro-topography in the presence of cAMP needs further investigation.

Following lesions of peripheral nerves, SCs reorganize to form longitudinal bands of Büngner (boB) which function as guides for regrowing axons, but their formation mechanism from a molecular point of view is unknown. A potential mechanism could be the polarized expression of adhesion proteins along the proximal–distal cell axis. Moreover, it was reported that placement of dissimilar adhesion characteristics in separate Schwann cell surface domains could aid longitudinal cell alignment [39]. Perhaps the reason why the SW10 cells remain round on the rougher substrates and they do not exhibit their typical bipolar or flattened morphology lies there.

It is worth stating here that by comparing the average number of cells that adhered on the different types of Si surfaces in the presence of a differentiation medium and in the cultures without a differentiation medium [11], we can deduce that the differentiation medium’s presence significantly reduces the adherence of the cells. This outcome is valid for both the mono- and the co-cultures. We already discussed how the presence of the differentiation medium influences the SW10 adherence. A similar mechanism leads to the reduced adhesion of N2a cells. The presence of a differentiation medium triggers the differentiation of N2a cells into having many properties of neurons and consequently the cells stop proliferating. This is the reason why the average number of N2a cells that adhere on the different types of Si surfaces is significantly smaller in the presence of a differentiation medium (RA or cAMP) than the number of cells adhering in the cultures without a differentiation medium.

In this study, we investigated for the first time the differentiation of N2a cells in co-culture with SW10 cells, both on the laser-patterned Si substrates and their replicas in PLGA. On the flat Si and PLGA, SW10 attached and proliferated well, while the N2a differentiated and developed long neurites. This is in accordance with other studies that have already reported the beneficial effect of glia for the differentiation of the neuronal cells in co-cultures of PNS cells. More specifically, an increase in neuronal NG108‐15 cell attachment when co-cultured with RN22 Schwann cells on a polyhydroxyalkanoate blend porous topography has been reported [40]. Moreover, a larger axonal length and area was observed in a co-culture of PC12 cells and SCs on polyethylene oxide (PEO) aligned nanofibres [41], while long neurites were observed in the presence of adipose‐derived stem cells differentiated to Schwann cells (dASCs) in a co-culture with dissociated dorsal root ganglion (DRG) neurons [42].

Nonetheless, as the roughness of the substrates was increasing, the N2a differentiation in co-culture was limited, despite the use of a differentiation medium. Taking into account our results concerning the N2a differentiation in mono-culture and the study of the SW10 culture with cAMP, this outcome might have been anticipated. In the mono-cultures with differentiation media the N2a cells remained round and did not differentiate on the medium and high roughness Si substrates. Moreover, in the presence of cAMP the SW10 cells remained round, as the roughness of the substrates increased. But, even so, the study of the N2a differentiation in co-culture with glia on the patterned Si substrates aims to discover if the presence of glia could potentially alter the N2a differentiation behaviour, since previous research had shown that the presence of glia can alter the N2a adhesion behaviour [11]. In this case, the presence of glia did not seem to act favorably on the differentiation of the neuronal-like cells and their differentiation behaviour was not affected, remaining the same as in the mono-culture. So, we can conclude that even if the adhesion behaviour of N2a cells changes in co-culture with glia [11], their differentiation behaviour does not alter. The impeding of the N2a differentiation in the co-culture can be attributed, as aforementioned, to the size of the patterns on both Si and PLGA substrates, which is smaller than the N2a dimensions, and can act as an inhibiting factor for the differentiation. This hypothesis can serve as a trigger for further investigations on the topological inhibition of the N2a differentiation and a protein screening is indispensable in order to show which pathways are followed in each case (i.e. on flat and rough substrates) and shed light on the exact mechanisms.

In order to answer the question of how the topography affects cellular responses at the molecular level, the activation state of major components of intracellular signaling pathways in N2a cells should be investigated. In this way, we expect to determine if the micro-conical Si substrates of increased roughness inhibit the neuronal cell differentiation by downregulating some specific protein pathways. This can be achieved via the development and application of functional proteomics approaches based in mass spectrometry. These results would lead to a better understanding of protein biochemical pathways and their role in the cell-micro/nanostructured interface.

To look at the bigger picture, an explanation on why topography is a crucial factor for neuronal differentiation could be related with the following molecular mechanism(s). Cells in living tissues are exposed to many physical signals imposed by neighbouring cells and the extracellular matrix (ECM). This includes ECM stiffness and viscoelasticity, but also its topology such as roughness, curvatures etc. Cellular responses such as adhesion and differentiation are affected by tissue stiffness [43]. Moreover, cells themselves have a characteristic stiffness, which is a result not only of their interaction with the surrounding microenvironment, but also of their biological and/or genetic status [44]. Mechanotransduction enables cells to sense and adapt to external forces and physical constraints [45]. In particular, variations of ECM stiffness or changes in cell shape caused by confining the cell’s adhesive area have a profound impact on cell behaviour across several cell types, such as stem cells and endothelial cells [46]. Moreover, the composition and the mechanical properties (stiffness) of a material play a significant role in the topography [47] and the wetting and morphological properties are also affected by the topography of the material.

That being the case, in our research we used two materials (Si and PLGA) with different chemical composition surface chemistry and stiffness, aiming at clarifying if the characteristics of the material could influence the topological inhibition of the N2a differentiation. Single crystal Si (100) has a Young’s modulus in the range of 125–200 GPa [48], while PLGA about 2 GPa [49]. Since we observed the same effect of the topography on the N2a differentiation, i.e. the differentiation is inhibited for both materials, we can deduce that the topography remains the main factor limiting the differentiation, even if the different stiffness may influence the cells growth in other terms, e.g. the neurite length of N2a cells in co-culture with SW10 was longer on the Si substrates than on their PLGA replicas.

In conclusion, in this study ultra-fast laser irradiation was applied in order to fabricate Si nano- and micro-structures with controlled geometry and pattern regularity. The Si structures were then replicated in PLGA and substrates of both materials were utilized as culture platforms to research the differentiation of N2a cells. The aim was to study the effect of three anisotropic discontinuous topographies (micro-spikes of different size) and an anisotropic continuous topography (nano-ripples) on the differentiation of neuronal-like cells in a mono-culture and in co-culture with glial cells. The topography of the substrates, both in Si and PLGA, was found to impede the N2a differentiation even in the presence of the differentiation medium. More specifically, the higher the roughness was, the more the differentiation limited. The present research also explored for the first time the differentiation of N2a cells in a co-culture with SW10 cells, coming to the same conclusion, i.e. N2a differentiation is inhibited as the substrate becomes rougher. These findings highlight the importance of the geometrical features of the substrate and how they can influence vital cell responses. Further investigation via protein screening methods can shed light on cells mechanotransduction, paving the way towards the development of biomaterial interfaces for neural implants in the field of regenerative medicine.

## Supplementary Information

Below is the link to the electronic supplementary material.

Figure S1 Phase contrast microscopy images of the 1+3 days N2a culture (6·10^4^ cells/ml) on TCP disks (A) without cAMP (control substrate) and (B) with 300μΜ cAMP. Confocal microscopy images of the 1+3 days N2a culture on the Si substrates (C) without cAMP on the flat Si substrate (control substrate), with 300μΜ cAMP on the (D) flat Si substrate, (E) nano-ripples, (F) low, (G) medium, (H) high roughness micro-cones and on the (I) TCP disk (control), (J) flat PLGA, (K) low and (L) high roughness PLGA replicas. In blue the cells nuclei stained with DAPI and in green the N2a neurites showing neuron-specific class III β-tubulin (stained with Tuj-1). (TIF 25333 kb)

Figure S2 Confocal images of the 1+3 days SW10 culture (6·10^4^ cells/ml) on (A) TCP disk and on (B) flat Si without differentiation medium (control substrates). On the flat Si substrates with: (C) 10μΜ RA, (D) 100μΜ cAMP, (E) 300μΜ cAMP, (F) 500μΜ cAMP. In blue the cells nuclei stained with DAPI and in red the actin filaments stained with rhodamine conjugated phalloidin. (TIF 2328 kb)

Figure S3 Numbers of SW10 cells cultured for 1+3 days with 300μΜ cAMP on: (A) flat Si, (B) nano-ripples, (C) low, (D) medium and (E) high roughness micro-cones. The results are expressed as cells/mm² ± standard error of the mean (SE). The data were subjected to ANOVA followed by Tukey test for multiple comparisons between pairs of means. The results were not statistically significant (p > 0.05). In blue the cells nuclei stained with DAPI and in red the actin filaments stained with rhodamine conjugated phalloidin. (TIF 748 kb)

Figure S4 Confocal images of the 1+3 days N2a and SW10 co-culture with 300μΜ cAMP on the: (A) rippled, (B) low, (C) medium and (D) high roughness Si substrates. In red the S100 positive SW10 cells, in green the N2a neurites showing neuron-specific class III β-tubulin (stained with Tuj-1) and in blue the cells nuclei stained with DAPI. These images are high-magnification views of the images presented in Figure 4E – H. (TIF 12566 kb)
