# Supplementary figures and images for: Laser-Structured Si and PLGA Inhibit the Neuro2a Differentiation in Mono- and Co-Culture with Glia
Source: Tissue Eng Regen Med. 2022 Dec 20;20(1):111–25. doi: 10.1007/s13770-022-00497-7 (PMC9852401; doi:10.1007/s13770-022-00497-7)

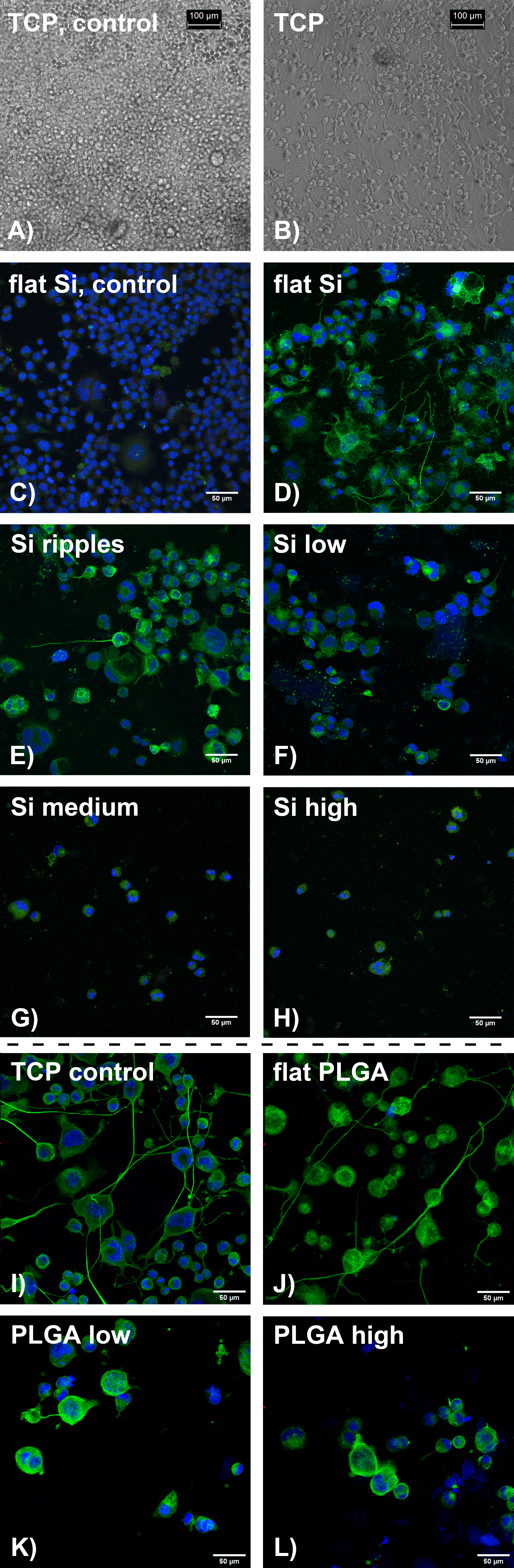

Supplement: Supplementary file 1 — Phase contrast microscopy images of the 1+3 days N2a culture (6·104 cells/ml) on TCP disks (A) without cAMP (control substrate) and (B) with 300μΜ cAMP. Confocal microscopy images of the 1+3 days N2a culture on the Si substrates (C) without cAMP on the flat Si substrate (control substrate), with 300μΜ cAMP on the (D) flat Si substrate, (E) nano-ripples, (F) low, (G) medium, (H) high roughness micro-cones and on the (I) TCP disk (control), (J) flat PLGA, (K) low and (L) high roughness PLGA replicas. In blue the cells nuclei stained with DAPI and in green the N2a neurites showing neuron-specific class III β-tubulin (stained with Tuj-1). (TIF 25333 kb) [file 13770_2022_497_MOESM1_ESM.tif]

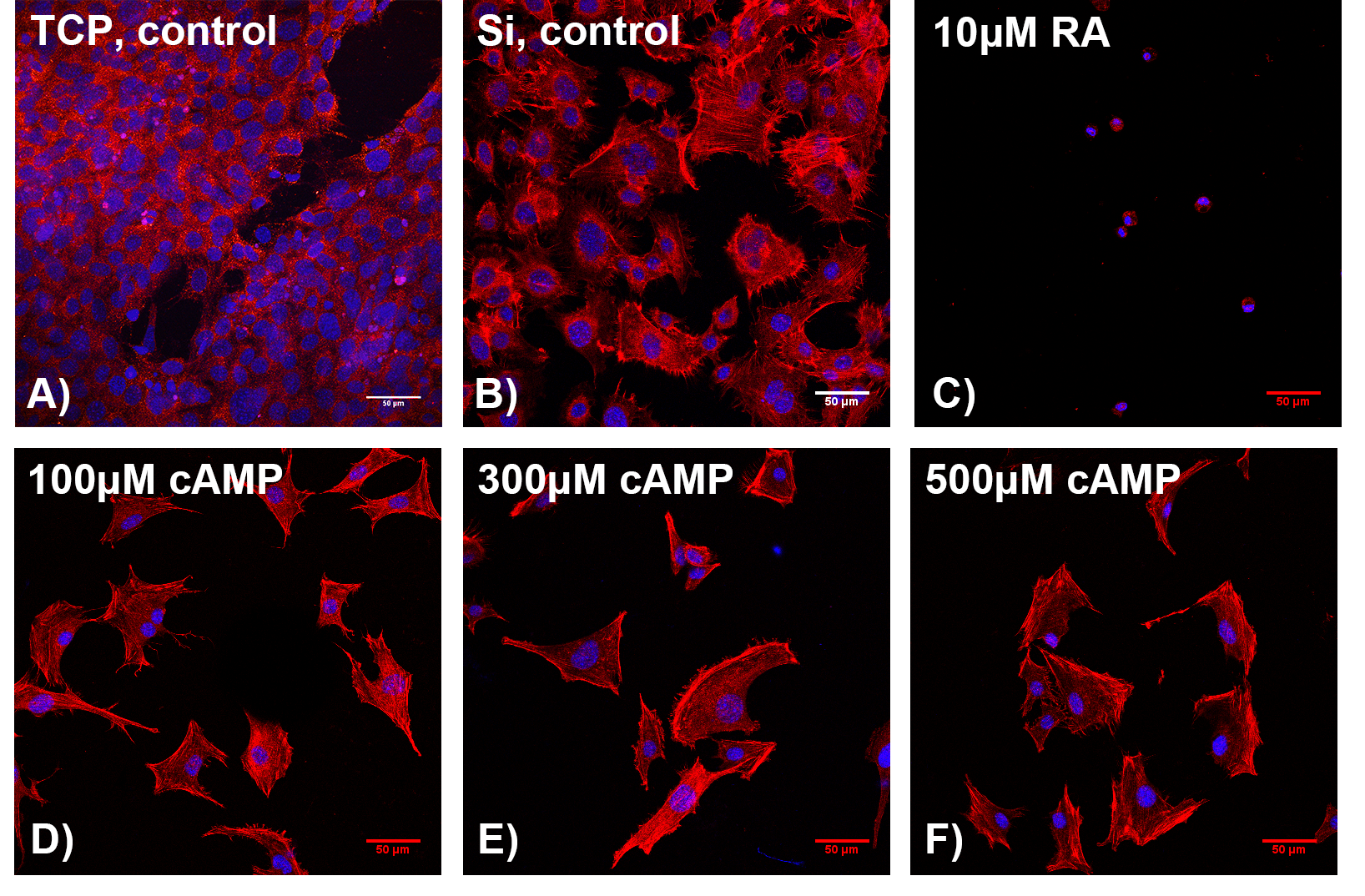

Supplement: Supplementary file 2 — Confocal images of the 1+3 days SW10 culture (6·104 cells/ml) on (A) TCP disk and on (B) flat Si without differentiation medium (control substrates). On the flat Si substrates with: (C) 10μΜ RA, (D) 100μΜ cAMP, (E) 300μΜ cAMP, (F) 500μΜ cAMP. In blue the cells nuclei stained with DAPI and in red the actin filaments stained with rhodamine conjugated phalloidin. (TIF 2328 kb) [file 13770_2022_497_MOESM2_ESM.tif]

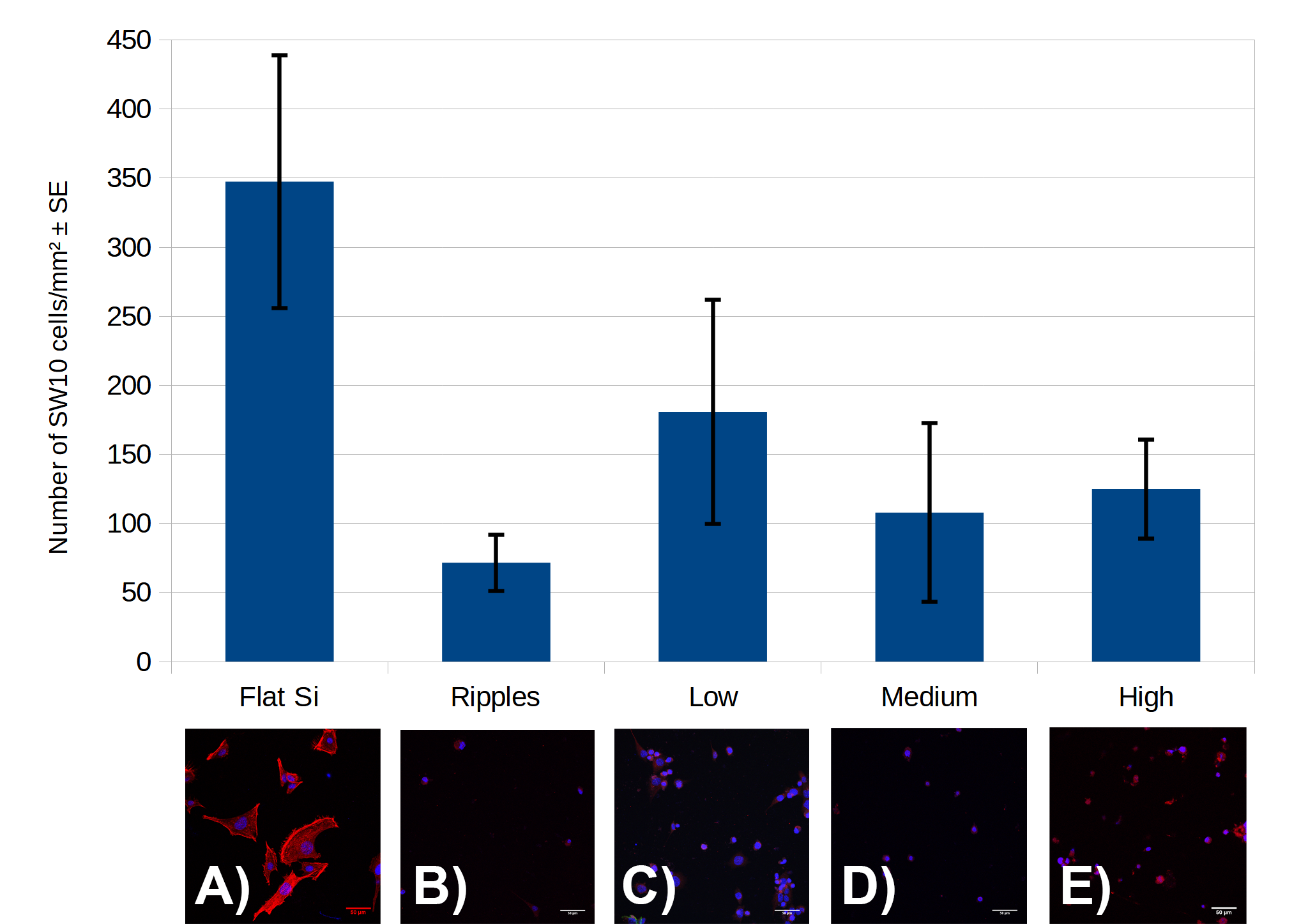

Supplement: Supplementary file 3 — Numbers of SW10 cells cultured for 1+3 days with 300μΜ cAMP on: (A) flat Si, (B) nano-ripples, (C) low, (D) medium and (E) high roughness micro-cones. The results are expressed as cells/mm² ± standard error of the mean (SE). The data were subjected to ANOVA followed by Tukey test for multiple comparisons between pairs of means. The results were not statistically significant (p > 0.05). In blue the cells nuclei stained with DAPI and in red the actin filaments stained with rhodamine conjugated phalloidin. (TIF 748 kb) [file 13770_2022_497_MOESM3_ESM.tif]

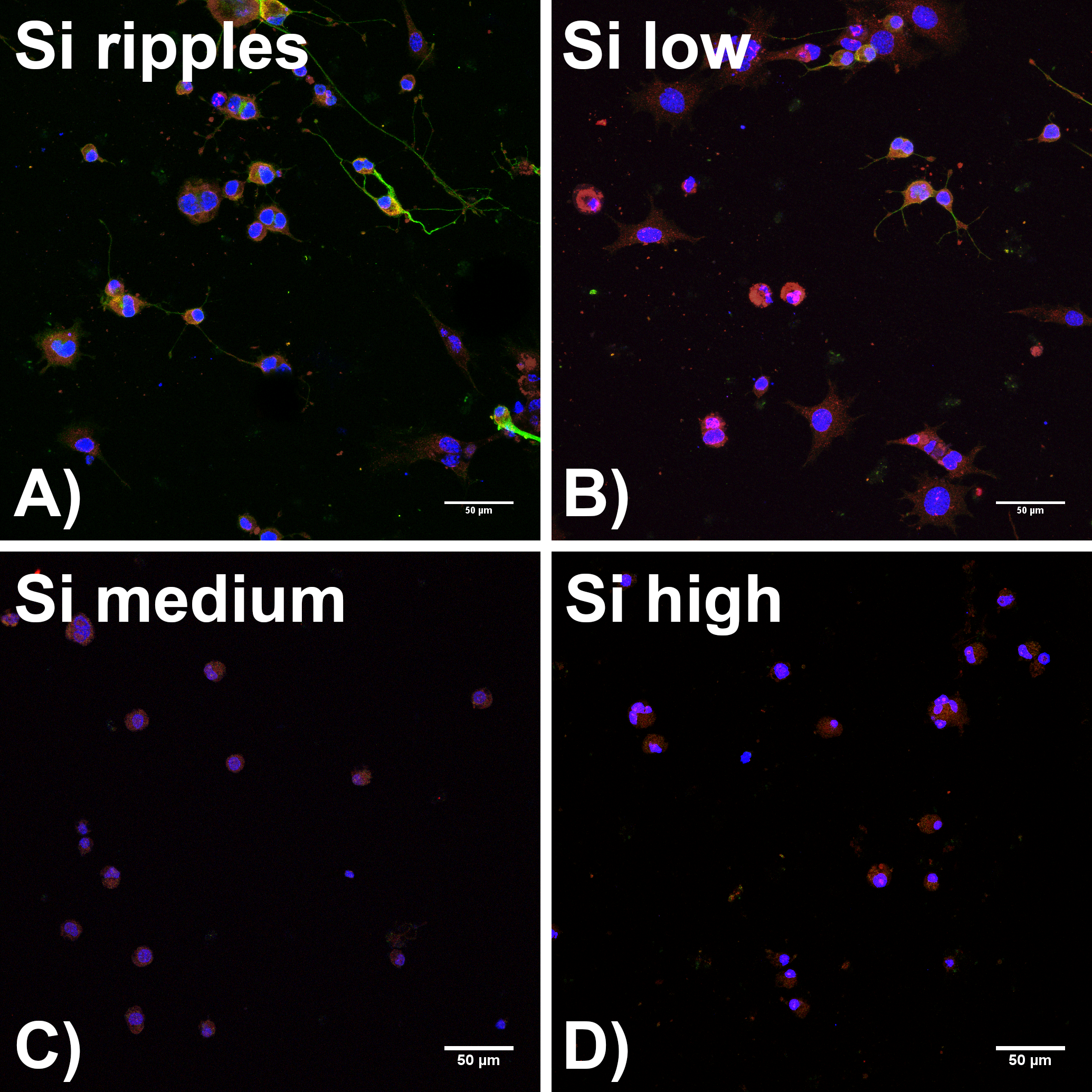

Supplement: Supplementary file 4 — Confocal images of the 1+3 days N2a and SW10 co-culture with 300μΜ cAMP on the: (A) rippled, (B) low, (C) medium and (D) high roughness Si substrates. In red the S100 positive SW10 cells, in green the N2a neurites showing neuron-specific class III β-tubulin (stained with Tuj-1) and in blue the cells nuclei stained with DAPI. These images are high-magnification views of the images presented in Figure 4E – H. (TIF 12566 kb) [file 13770_2022_497_MOESM4_ESM.tif]
